# Transcriptomic Profiling of Toxic Copper Overload Induced by CuO Nanoparticles or Copper Ions in Human Lung Epithelial and Liver Cells

**DOI:** 10.3390/nano16100590

**Published:** 2026-05-12

**Authors:** Jana Kuhn, Anda R. Gliga, Cheyenne Ines Aissouni, Anna Maria Glowacki, Marlene Parsdorfer, Martin Link, Hanna Lovisa Karlsson, Andrea Hartwig

**Affiliations:** 1 Department of Food Chemistry and Toxicology, Institute of Applied Biosciences (IAB), Karlsruhe Institute of Technology, 76131 Karlsruhe, Germany; 2 Unit of Metals and Health, Institute of Environmental Medicine, Karolinska Institutet, 171 77 Stockholm, Sweden; 3 Department of Pharmaceutical Biosciences, Uppsala Universitet, 751 24 Uppsala, Sweden

**Keywords:** nanotoxicology, in vitro research, copper, transcriptomics

## Abstract

The transition metal copper (Cu) is an essential trace element for humans and serves as a cofactor for numerous enzymes. Therefore, intracellular Cu homeostasis must be tightly regulated. Meanwhile, Cu is increasingly used in industrial and biomedical applications, particularly in nanoparticle (NP) form. However, studies have demonstrated that Cu(II) oxide (CuO) NPs are highly toxic. Therefore, understanding the underlying toxic effects of such compounds is of the utmost importance. In this context, transcriptomic profiling is regarded as a valuable tool. Nevertheless, comparative studies addressing organ-relevant models, such as the liver and lungs, are scarce. Furthermore, no transcriptomic studies have been conducted on human bronchial lung epithelial cells exposed to CuO NPs and Cu^2+^. In this study, we compared the cellular effects of human bronchial lung epithelial cells exposed to both CuO NPs and Cu^2+^ to the effects in human liver cells exposed to Cu^2+^ by applying RNA sequencing. Although cytotoxicity was comparable, we showed that Cu uptake was highly dependent on both the cell type and the form of Cu. The most pronounced concentration-dependent transcriptional changes were observed with CuO NP exposure in BEAS-2B cells. The only differentially expressed genes (DEGs) found by all exposures and treatments were metallothioneins (MTs). The most sensitive targets of Cu-induced toxicity were related to nuclear factor erythroid 2-related factor 2 (NRF2), nuclear factor kappa-light-chain-enhancer of activated B cells (NFkB), and mitogen-activated protein kinase (MAPK) signaling. Furthermore, the effects observed at the transcriptome level were studied at the functional level, such as cell cycle regulation and cytokine release. Thus, we demonstrated that the two cell types differ in susceptibility, and that complementing transcriptome profiling with functional studies provides important mechanistic insights.

## 1. Introduction

Cu is an essential trace element for the human body, yet it can be toxic when levels exceed homeostatic control. As a redox-active metal, Cu takes part in multiple relevant physiological processes, including energy metabolism, antioxidant defense, iron (Fe) metabolism, collagen synthesis, and neurotransmitter synthesis. For instance, Cu serves as a cofactor for mitochondrial cytochrome c oxidase, Cu/Zn superoxide-dismutase (SOD), which is involved in antioxidant defense, and for lysyl oxidase, a key enzyme in collagen synthesis [1,2]. Therefore, Cu is involved in the redox chemistry of various proteins. However, its homeostatic regulation can be overwhelmed by factors such as Cu overload. This can occur through unintended uptake due to environmental pollution, in occupational settings, or via disturbed uptake/excretion mechanisms [3,4,5]. Typically, Cu is bound to metalloproteins, such as MTs or glutathione (GSH), to prevent unwanted reactivity [1]. Unbound Cu(I) can participate in Fenton-like reactions. These reactions generate reactive oxygen species (ROS), in terms of highly reactive hydroxyl radicals, which cause oxidative stress to cells [6]. Growing concerns regarding the potential health hazards of Cu are based on its increasing use, both in industry and biomedical applications. In this context, increased production of Cu-containing nanomaterials is receiving more attention. From 2010 to 2025, the global CuO NP production was estimated to increase from 200 t to 1600 t [7,8]. Simultaneously, CuO NPs have been shown to be significantly more toxic than microparticulate CuO or Cu ions [9,10]. The liver is primarily affected by Cu overload, because it is the main storage site for Cu in the human body. This is particularly important for Cu exposure via food or drinking water, as Cu first enters the gastrointestinal tract and subsequently enters the enterohepatic circulation. Up to 75% of dietary Cu can be absorbed in the gastrointestinal tract. Absorbed Cu ions are transported to the liver, where they are distributed to other organs or excreted through the bile [1]. In occupational settings, however, the main target organ of CuO NPs is the lung, because inhalation is considered the primary exposure route. Accumulation of CuO NPs in the liver is considered less relevant, because the entry of highly soluble CuO NPs into the gastrointestinal tract would result in ion release under acidic conditions in the stomach [11]. Therefore, the liver would be exposed to Cu ions that enter the enterohepatic circulation instead of CuO NPs themselves.

Despite the growing body of research on Cu-induced toxicity, mechanistic comparisons of CuO NPs and Cu^2+^ in cell cultures representing primary target organs remain scarce. To date, only few transcriptomic investigations of Cu-induced toxicity have been conducted in either lung cell models exposed to Cu-containing nanomaterials or in liver cell models exposed to Cu^2+^. Boyadzhiev and co-authors studied the global transcriptome of CuO NPs in mouse epithelial lung cells, and found thousands of DEGs after 24 h of exposure to 25 µg/mL CuO NPs. Canonical pathway analysis revealed the strongest effects on pathways related to phagosomes and Fe homeostasis. Other significantly induced pathways were related to the cellular stress response. In comparison, 7 µg/mL CuCl_2_ did not alter pathway signaling after 24 h [12]. To the best of our knowledge, data on the global transcriptome profiling of Cu in human epithelial lung cells is limited to a study of A549 cells. In that study, it was demonstrated that genes related to the cellular stress responses and metabolic processes were enriched upon exposure to CuO NPs. Furthermore, induction of MTs was one of the most sensitive responses of A549 cells exposed to Cu, and a substantial effect on the cell cycle regulation was reported [13]. However, A549 cells are lung tumor cells, with NRF2 constitutively active [14]. Regarding studies on human bronchial epithelial cells, only high-throughput gene expression studies of a limited number of genes of interest have been published. One such study is that of Strauch et al. [9]. In their study, the toxicity of CuO NPs and Cu ions was compared with an equivalent dose of Cu in BEAS-2B cells. The results showed that CuO NPs particularly induced genes related to metal homeostasis, oxidative stress, and cell cycle regulation. Moreover, *IL8* expression was massively increased [9]. Several transcriptomic studies have examined the effects of Cu^2+^ in hepatocytes, which serve as major Cu storage cells. These studies reported that Cu induces the activation of genes involved in metal homeostasis and oxidative stress, as well as broader regulators of gene expression. In particular, higher exposure doses of Cu were stated to induce effects on MAPK and NFkB inflammatory pathways [15,16]. Muller and co-authors demonstrated comparable results in HepG2 cells. Unlike the other studies, the cells were exposed to CuO NPs without serum [17]. Overall, the induction of oxidative stress was considered an underlying mechanism of Cu toxicity. However, to the best of our knowledge, there are no transcriptomic studies that directly compare cellular responses to Cu-induced toxicity in organ-relevant human cell models. Furthermore, studies typically focus solely on transcriptomic profiling. However, cellular fate at the functional level can differ. Thus, it is crucial to include additional endpoint studies to gain a more comprehensive understanding of Cu toxicity at the mechanistic level. Thus, we performed global transcriptome profiling to investigate the effects of CuO NPs and Cu^2+^ on human bronchial epithelial cells (BEAS-2B) after 24 h of acute exposure. In parallel, we exposed human hepatoblastoma cells (HepG2) to Cu^2+^ to enable comparison with a liver model. In addition to assessing the cytotoxicity and cellular uptake of the Cu compounds, we studied cell cycle regulation, cytokine release, and the potential induction of ROS by NPs on a functional level. Thus, we provide insights into the comparative transcriptomic profiling of Cu overload across organ-relevant human cell models, and put them into context with their respective endpoints.

## 2. Materials and Methods

### 2.1. Cell Culture and Reagents

Human bronchiolar BEAS-2B cells were kindly provided by PD Dr. Carsten Weiss (Karlsruhe Institute of Technology, KIT). These cells were originally obtained from the American Type Culture Collection (ATCC, CRL-3588) and cultured in keratinocyte growth medium (KGM) cell culture medium, which was supplemented with KGM SingleQuots (Lonza, Basel, Switzerland). Prior to the initiation of the cultivation process, tissue culture flasks or plates were pre-coated with a 1:1:1 mixture of 10 µg/mL fibronectin, 30 µg/mL collagen, and 10 µg/mL bovine serum albumin for 30 min at 37 °C. The cells were cultivated in T75 tissue culture flasks and passaged once a week. Following the removal of the medium, the cells were washed with phosphate-buffered saline (PBS) that had been warmed to 37 °C. Thereafter, the cells were treated with 2 mL Accutase^®^ for 3 min in a humidified incubator. The cell suspension was then used for subsequent sub-cultivation.

HepG2 cells were obtained from ATCC (HB-8065) and cultivated in Dulbecco’s modified Eagle medium (DMEM, Gibco, Waltham, MA, USA) supplemented with 10% fetal bovine serum (FBS) and 2% penicillin/streptomycin. The cells were passaged twice weekly in T75 tissue culture flasks. After washing cells with PBS, the cells were subjected to a 0.25% trypsin-EDTA treatment (in PBS) for 3 min in a humidified incubator. Subsequently, the enzyme activity was terminated by the addition of cell culture medium supplemented with 10% FBS. Subsequently, the cell suspension was utilized for subcultivation. Both cell lines were cultivated as submerged monolayers and maintained at 37 °C and 5% CO_2_ in a humidified incubator. In order to obtain cell pellets following exposure experiments, the same procedure as described here was applied, with adapted volumes. Cell suspensions were transferred to 1.5 mL microcentrifugation tubes, centrifuged, and washed with 4 °C cold PBS. IRB statement: As this study utilized exclusively established commercialized cell lines, it did not require institutional review board approval.

### 2.2. Copper Compounds

CuO NPs were kindly provided by Wendel Wohlleben (BASF, Ludwigshafen, Germany), and were manufactured by PlasmaChem (Berlin, Germany, LOT YF1311107). They were processed in accordance with the established NanoGenoTox protocol (Chapter 2.2, following liquid dispersion with ethanol pre-wetting and BSA-water) [18]. Frozen aliquots were thawed for 1 min at 60 °C in an ultrasonic water bath. The initial characterization of CuO NPs was conducted by Wall et al. [19]. Cu^2+^ was utilized in the form of CuCl_2_*2H_2_O (Sigma-Alrich, St. Louis, MO, USA). A stock solution of 100 mM was prepared in water and subsequently sterile filtered prior to the execution of exposure experiments. Both compounds were tested negative for endotoxin content consistent with principles of endotoxin-free experimentation. This was achieved by conducting a limulus amebocyte lysate (LAL)-endotoxin assay (Pierce Chromogenic Endotoxin Quant Kit, Thermo Fisher Scientific, Waltham, MA, USA) according to the manufacturer’s instructions.

### 2.3. Cytotoxicity

For the determination of cytotoxicity, the CellTiter-Glo^®^ Luminescent Cell Viability Assay Kit (Promega, Madison, WI, USA) was applied, quantifying the ATP content as a measure of cell viability. The manufacturer’s instructions were adhered to during the process. Briefly, 3 × 10^4^ cells/100 µL were seeded in a white 96-well plate with a transparent bottom for 24 h. Thereafter, the cells were exposed to 0–120 µg/mL Cu for 24 h in triplicate. Subsequently, the cells were washed with 100 µL PBS per well, and 60 µL of cell culture medium was added. Following a 30 min equilibration period at room temperature, 60 µL of the assay mix solution was added to each well. After shaking the plate for 2 min in orbital shake mode, the bioluminescent signal was determined after an additional 10 min using a Tecan Infinite^®^ 200 PRO plate reader (Tecan Group Ltd., Männedorf, Switzerland). Notably, all exposures, controls, and medium controls were also measured in duplicates in cell-free wells. This was done to rule out any potential interference with the assay. The ATP content was then normalized to untreated controls and expressed as a percentage.

### 2.4. Copper Uptake

The quantification of cellular Cu uptake was executed through the implementation of graphite furnace atomic absorption spectroscopy (GF-AAS) (PinAAcle 900T, Perkin Elmer, Waltham, MA, USA) with a Cu hollow cathode lamp, argon as protectant gas, and a 0.7 nm slit. Initially, 1 × 10^6^ cells were seeded in 4.2 mL medium in 6 cm plates for 24 h. Thereafter, the cells were exposed to 0–40 µg/mL Cu for 4–24 h. Subsequently, the cells were counted with a Casy^®^ TT Cell Counter (Omni Life Sciences, Bremen, Germany). The mean diameter of the cells was also obtained. Afterwards, the cells were pelleted and dissolved in 500 µL of a 1:1 mixture of 69% HNO_3_:30% H_2_O_2_, which was used for oxidative digestion. This was complemented by temperature-based evaporation of the matrix compounds. Then, the obtained Cu samples were dissolved in 500 µL of 0.2% HNO_3_. A standard calibration curve between 0 and 25 µg/L Cu was prepared, employing a defined Cu standard (Carl Roth, Karlsruhe, Germany). All measurements were executed in triplicate, with 20 µL/analysis. Moreover, 5 µL of a matrix modifier composed of 0.005 mg palladium and 0.003 mg magnesium was added to each replicate. The application of blind value samples yielded a decision limit of 0.261 µg/L, a detection limit of 0.522 µg/L, and a determination limit of 1.087 µg/mL, as determined by calculations in accordance with DIN32645. The following temperature program was applied: 120 °C for 30 s, 140 °C for 30 s with 15 s ramp, pyrolysis at 1200 °C for 20 s with 10 s ramp, atomization at 2000 °C for 5 s, and bake out at 2450 °C for 3 s with 1 s ramp. Cu concentration per sample was obtained blank-corrected in µg/L and thereafter determined as the mean Cu content per cell in µM. This calculation was based on cell volume and mean Cu mass per cell.

### 2.5. Transcriptome Analysis

RNA isolation was carried out using the NucleoSpin RNA Plus Kit (Marchery Nagel, Düren, Germany). A total of 4.5 × 10^5^ cells/1.88 mL were seeded in 6-well plates and maintained for 24 h. Thereafter, the cells were exposed to 0–40 µg/mL Cu for 24 h. BEAS-2B cells were exposed to both CuO NPs and CuCl_2_, whereas HepG2 cells were exposed exclusively to Cu^2+^. After exposure, the duplicates prepared per treatment condition were pooled. The isolation of RNA from the obtained cell pellets was performed according to the manufacturer’s instructions. In summary, cell pellets were resuspended in 350 µL lysis buffer and subsequently transferred to gDNA removal columns. Placed in collection tubes, samples were then spun down at 11.000 *g* for 30 s. Afterwards, 100 µL binding solution was added to the flow-through, and the mixture was transferred to an RNA-binding column placed in a collection tube. After centrifugation at 11.000 *g* for 20 s, RNA bound to the column was washed three times. Notably, centrifugation was always performed with these parameters, unless stated otherwise. Subsequently, 200 µL wash buffer 1, 600 µL wash buffer 2, and 250 µL wash buffer 2 were added to the column, which was centrifuged following the addition of each wash buffer. Thereafter, RNA elution was executed by the addition of 30 µL RNAse-free water and subsequent centrifugation at 11.000 *g* for 1 min. To increase the yield, the eluate was re-applied to the column and again centrifuged. Isolated RNA concentration was quantified by using a photo spectrometer (NanoDrop™ One, Thermo Fisher Scientific, Waltham, MA, USA) at 260 nm absorbance.

RNA sequencing was facilitated by NovoGene (Munich, Germany) with an Illumina NovaSeq sequencer (Illumina, San Diego, CA, USA) and a PE150 platform. In addition, NovoGene supplied total read counts, which were analyzed in relation to the reference genome Homo Sapiens [GRCh37]. For each treatment, three independent experiments were analyzed.

Raw data and further information can be found on ArrayExpress (accession number E-MTAB-16794).

The Bioconductor package DESeq2 (version 3.22) for the R programming language (version 4.4.2.) was used for differential gene expression analysis [20]. Consequently, only significantly changed genes compared to untreated controls with an FDR-adjusted *p*-value ≤ 0.05 were identified as DEGs. IPA software (version 145030503, Ingenuity Systems, Redwood City, CA, USA) was applied for canonical pathway enrichment analysis and upstream regulator analysis on all DEGs that had an absolute log_2_ fold change > 0.5. This threshold was chosen due to the high number of DEGs found in BEAS-2B cells treated with CuO NPs. Therefore, the threshold enabled us to cover all major effects considered relevant. Venn diagrams were prepared using the online tool Venny (version 2.1.0) [21]. Additionally, a gene set enrichment analysis with KEGG pathways as a functional database was performed with a ranking list including Ensembl gene identifiers and log_2_ fold change values in WEBGESTALT (version 2024) [22].

### 2.6. DCFH-DA Assay

The oxidative potential of CuO NPs was determined by an acellular DCFH-DA assay excluding the addition of HRP, as previously described by Kessler et al. [23]. Briefly, 0–100 µg/mL CuO NPs and 100 µg/mL NiO NPs were diluted in PBS. NiO NPs served as a positive control. The DCFH-DA probe was cleaved to DCFH by 0.01 M NaOH for 30 min. The reaction was terminated by dilution with PBS. A sample volume of 25 µL was added to each well in a black 96-well plate with a transparent bottom. Samples were analyzed in triplicate. This was followed by the addition of 100 µL PBS per well. Then, 75 µL DCFH was added to each well. Non-DCFH controls and 40 µg/mL Cu^2+^ were also analyzed. The fluorescent signal was obtained at 485/530 nm excitation/emission after 18 min with a Tecan Infinite^®^ M200 PRO (Tecan Group Ltd., Männedorf, Switzerland). Baseline correction was performed by subtracting non-DCFH control values from sample values, and mean values per treatment condition were normalized to mean values of untreated control samples.

### 2.7. Cell Cycle Control by Flow Cytometry

In this experiment, 1 × 10^6^ BEAS-2B cells were seeded in 4.2 mL medium in 6 cm plates, and 1.16 × 10^6^ HepG2 cells were seeded in 5 mL medium in T25 flasks for 24 h. The cells were then exposed to 0–40 µg/mL Cu by both CuO NPs and CuCl_2_ for 24 h, with each condition in duplicate. Subsequent to the termination of exposure, 1 × 10^6^ cells/1 mL PBS were fixed by the drop-wise addition of 3 mL 96% ethanol to the cell suspension, under continuous mixing, in a 10 mL round-bottom glass, followed by storage at −20 °C overnight. The fixed cells were centrifuged at 3220 g for 4 min and at 4 °C. Subsequently, cell pellets were incubated in CyStain^®^ DNA staining solution (Partec, Görlitz, Germany) for 30 min on ice, light-protected. The fluorescent signal was obtained using a BD LSRFortessa flow cytometer (BD Biosciences, Franklin Lakes, NJ, USA) at 488/425–475 nm excitation/emission. The cell count was then plotted against the fluorescent signal in a histogram, which was gated to obtain data on G_0_/G_1_-phase, S-phase, and G_2_/M-phase.

### 2.8. Cytokine Release Immunoassay

A 4-plex immunoassay (V-Plex Human Proinflammatory Panel II) was used to investigate three pro-inflammatory cytokines: IL1ß, IL6, and TNFα. Meanwhile, IL8 was investigated with a one-plex immunoassay (V-Plex Human IL-8). The two assays were both based on electrochemiluminescent assays that had been purchased from MSD (Meso Scale Discovery, Rockville, MD, USA). The assay was performed in accordance with the manufacturer’s recommended workflow. In brief, 1.5 × 10^4^ cells/100 µL/well were seeded in 96-well plates for 24 h and subsequently exposed to both Cu compounds for 24 h in triplicate. Subsequently, 50 µL of the resulting supernatant from each well was collected, and the triplicates were then amalgamated. Potentially remaining contaminants, such as NPs, were eliminated by centrifugation at 300 *g* for 5 min. Consequently, supplementary medium controls were meticulously prepared. Samples were diluted in constituted Diluent 2 at a ratio of 1:2, and the V-Plex plate provided by the manufacturer was washed three times with 150 µL/well 1× wash buffer. In this experiment, 50 µL sample/well, standard, or control was added to the plate in triplicate, and incubated for 2 h. The plate was then washed three times with 150 µL/well 1× wash buffer. Following this, 25 µL/well of respective detection antibodies were added and incubated for 2 h. After a final washing step for three times, 150 µL/well 2× read buffer was added. Cytokine signal was measured using an MSD plate reader. The experimental sample values were extrapolated from the standard curve that had been obtained. Mean values for each treatment condition were normalized to the mean values of untreated controls.

### 2.9. Statistical Analysis

Each analysis was performed with three independent experiments. The program GraphPad Prism Software (version 10.6.1) was used for the statistical analysis of all data with the exception of RNA sequencing, for which the statistical program R (version 4.4.2.) and Ingenuity Systems’ IPA software (license from Ingenuity Systems, Redwood City, CA, USA) were utilized. The GraphPad Prism software (version Prism 10.6.1) was used to implement a one- or two-way analysis of variance followed by Dunnett’s multiple comparison test. GraphPad Prism was further employed for the graphical representation of the data, which is depicted as the mean of all independent experiments. In the context of bar charts, the standard error of the mean (sem) is also illustrated.

## 3. Results

This study compared the impact of CuO NPs and Cu ions on BEAS-2B cells, which was further compared to Cu ion-induced toxicity in HepG2 cells using RNA-sequencing. First, the cytotoxicity and cellular uptake of all treatments were studied. Moreover, we compared the effects of Cu exposure at the functional level based on transcriptomic data. These endpoints included cell cycle regulation, inflammation, and ROS induction.

### 3.1. Cell Viability

**Figure 1:**
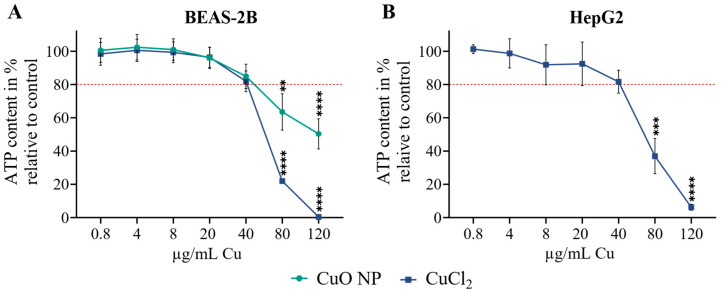
BEAS-2B cells (**A**) were exposed to CuO NPs and CuCl_2_, and HepG2 cells (**B**) were exposed to CuCl_2_ for 24 h. Cell viability was determined by an ATP assay. The red dashed line shows the minimum level of cell viability at which a concentration was considered to have low cytotoxicity. Results are depicted as the mean of three independent experiments ± sem. An ordinary two-way ANOVA was applied for the BEAS-2B cells, and an ordinary one-way ANOVA for HepG2 cells, both followed by a Dunnett’s multiple comparison test: ** *p* ≤ 0.01, *** *p* ≤ 0.001, **** *p* ≤ 0.0001.

Cell viability was assessed to determine non- and low cytotoxic concentrations of both Cu compounds for further experimentation. Low cytotoxic concentrations were considered doses of Cu that led to a maximum decline to 80% in cell viability. As Figure 1 shows, neither CuO NPs nor CuCl_2_ exhibited cytotoxicity up to 20 µg/mL Cu. A low cytotoxic concentration of 40 µg/mL Cu was found in both cell lines. Thus, 4–40 µg/mL Cu were chosen as the exposure concentration for subsequent experiments. Notably, the results on cytotoxicity were in good agreement with previous studies [16,19].

### 3.2. Copper Uptake

**Figure 2:**
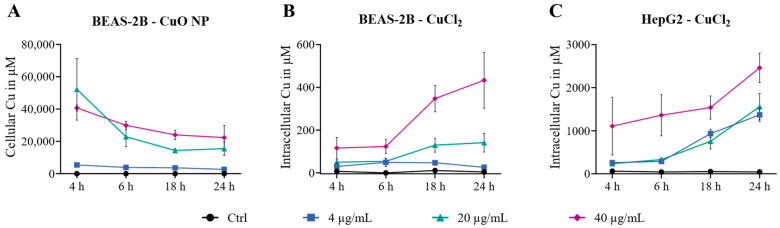
BEAS-2B cells were exposed to CuO NPs (**A**) and CuCl_2_ (**B**), and HepG2 cells were exposed to CuCl_2_ (**C**) for 4–24 h. Cellular uptake was measured by GF-AAS. Results are depicted as the mean cellular Cu content in µM per cell of three independent experiments ± sem.

The uptake of Cu into cells, as well as the remaining CuO NPs attached to the cell membrane after washing, was measured by GF-AAS between 4 h and 24 h. As shown in Figure 2, BEAS-2B cells exhibited the highest uptake of CuO NPs, reaching up to 52,000 µM Cu per cell after 4 h, which was the earliest time point measured. Rapid uptake was observed after 4 h, and cellular Cu levels decreased concentration-dependently to a maximum of 25,000 µM after 24 h. A clear accumulation of Cu in the lung cells was observed related to the maximum exposure concentration of 40 µg/mL Cu, which equals 628 µM (see Table 1). In comparison, the situation was different regarding the uptake of CuCl_2_ in both cell lines. BEAS-2B cells took up Cu ions time- and concentration-dependently, reaching maximum intracellular Cu levels of 420 µM after 24 h. Thus, the cellular Cu load in BEAS-2B cells exposed to CuCl_2_ was up to two orders of magnitude lower than in cells exposed to CuO NPs. HepG2 cells exposed to CuCl_2_ exhibited time-dependent uptake, reaching levels of up to 2460 µM. Therefore, also in HepG2 cells exposed to Cu ions, an intracellular accumulation of Cu was evident. Notably, the cellular Cu levels in HepG2 cells were more than five times higher than in BEAS-2B cells exposed to CuCl_2_. These results demonstrate significant differences in cellular Cu uptake between the two Cu compounds and cell types. It is important to note that the membrane fraction of lung cells exposed to CuO NPs was not separated. Therefore, also CuO NPs that remained attached to the cell membrane after washing contributed to cellular Cu levels measured by this approach. In a previous study by our group using the same cell line, 3.5-fold higher values were found by this approach when compared to intracellular Cu levels after removal of the outer membrane [9]. Even after correcting for this potential overestimation, the intracellular Cu load in BEAS-2B cells exposed to CuO NPs within this study was still much higher than in cells exposed to Cu ions.

### 3.3. Transcriptome Analysis

**Figure 3:**
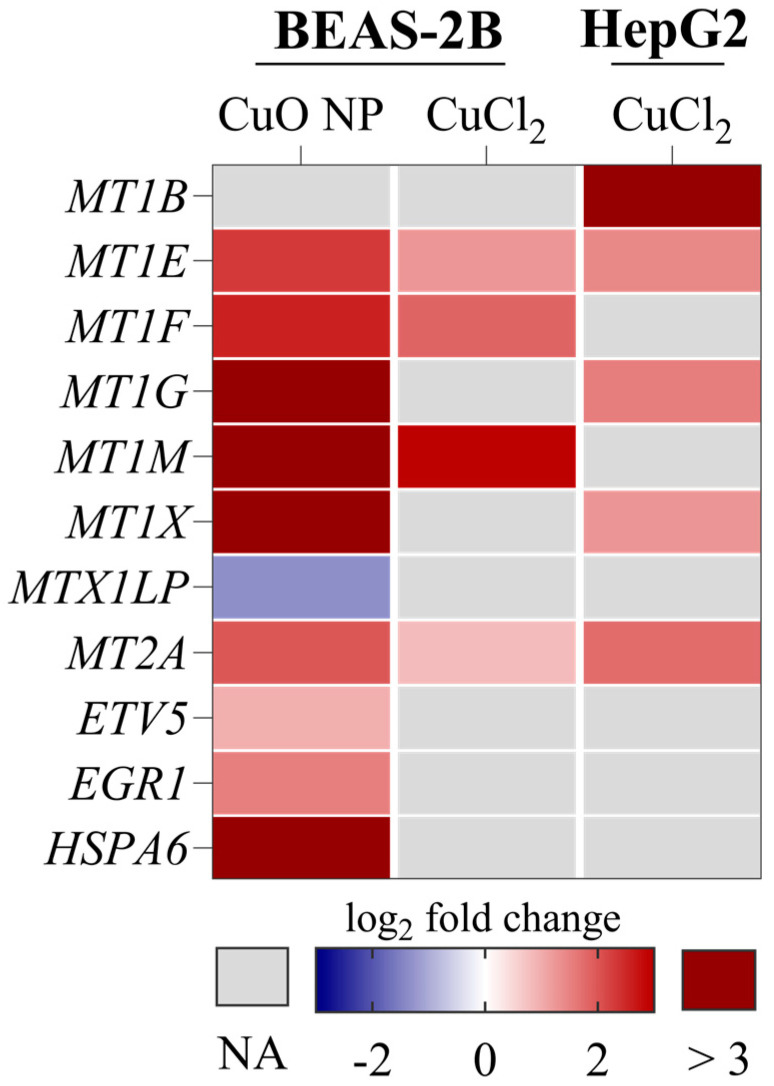
Overlapping DEGs between all exposure concentrations per treatment and cell line. Genes are depicted as log_2_ fold change in the lowest exposure dose of 4 µg/mL Cu, compared to untreated controls of three independent experiments. NA = value not available.

**Figure 4:**
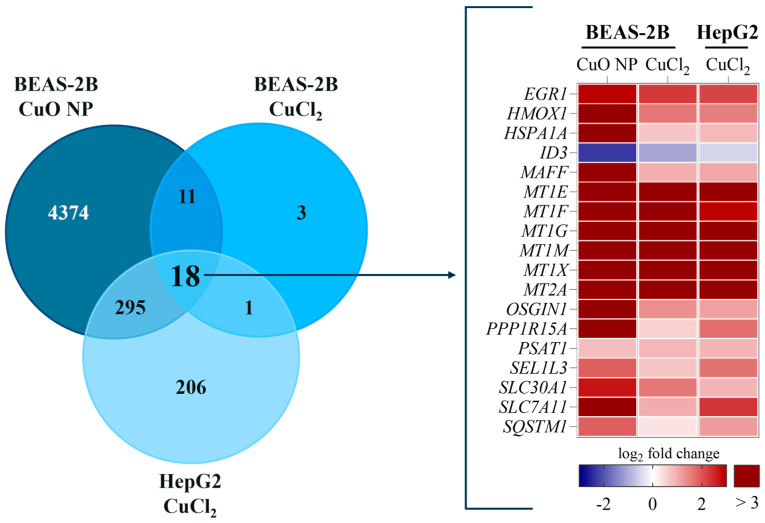
Overlapping DEGs found in both cell lines exposed to 40 µg/mL Cu. The Venn diagram shows the number of DEGs identified by Ensembl IDs per highest exposure condition in each treatment. A total of 18 DEGs were identified as being shared by all treatments. In the heatmap, these 18 overlapping DEGs are depicted as log_2_ fold change compared to untreated controls of three independent experiments.

We investigated the global transcriptomes of BEAS-2B and HepG2 cells exposed to Cu using RNA sequencing. As shown in Table 1, exposure to CuO NPs resulted in the highest number of DEGs in BEAS-2B cells. A total of 4698 DEGs were identified at the highest exposure concentration. By comparison, 520 DEGs were found in HepG2 cells exposed to 40 µg/mL CuCl_2_, and only 33 DEGs were found in BEAS-2B cells exposed to CuCl_2_. Thus, the number of DEGs reflected the cellular Cu load, as measured by uptake studies. Table 2 also shows that upon all exposures, a clear concentration-dependent increase in DEGs occurred with all exposures. Interestingly, the lowest exposure concentration had almost no effect on changes in gene expression, regardless of the Cu compound or cell type studied. Figure 3 lists overlapping DEGs between the different Cu concentrations per treatment. As can be seen, the only DEGs shared by all exposure treatments and concentrations were the MTs. At the lowest Cu concentration, *MT1E* and *MT2A* overlapped among all treatments. Regarding the concentration-dependent increase in DEGs upon Cu exposure, a marked increase in DEGs was particularly observed for the highest exposure concentration. In contrast, the increase in DEGs upon exposure to 20 µg/mL CuCl_2_ was limited in both cell lines. Figure 4 shows all DEGs shared by all treatments at the highest Cu exposure dose. Next to MTs, most of the overlapping DEGs were associated with the cellular (oxidative) stress response, including *heme oxygenase 1* (*HMOX1*), *heat shock protein family A member 1A* (*HSPA1A*), *MAF bZIP transcription factor F* (*MAFF*), *oxidative stress induced growth inhibitor 1* (*OSGIN1*), *protein phosphatase 1 regulatory subunit 15A* (*PPP1R15A*), *SEL1L family member 3* (*SEL1L3*), *solute carrier family 7 member 11* (*SLC7A11*), and *sequestosome 1* (*SQSTM1*). Therefore, cellular stress is considered a primary feature of Cu-induced toxicity. Other shared DEGs were associated with transcriptional regulation (*early growth response 1* (*EGR1*), *inhibitor of DNA binding 3* (*ID3*)), proliferation (*phosphoserine aminotransferase 1* (*PSAT1*)), and Zn transport (*transmembrane protein 163* (*SLC30A11*)).

**Figure 5:**
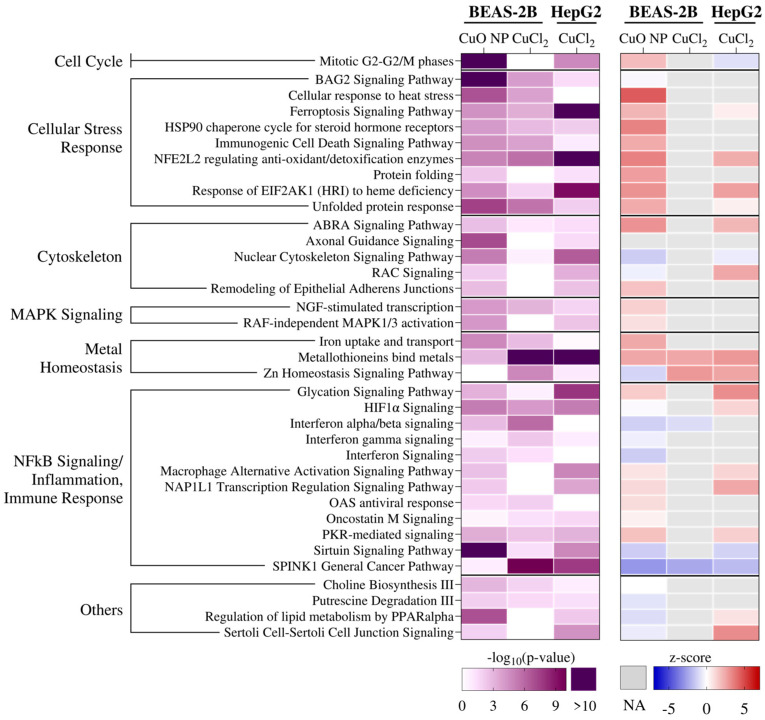
Canonical pathway enrichment analysis regarding the highest exposure concentration of 40 µg/mL Cu in BEAS-2B and HepG2 cells after 24 h exposure. The table shows all canonical pathways that were significantly enriched in at least two treatments in at least two concentrations. The level of significance is represented by the FDR-corrected −log_10_(*p*-value), whereby any value greater than 1.3 was considered significant, depicted in purple. The z-score further provides information on pathway activation, whereby positive values (red) are predictive of pathway activation and negative values (blue) are predictive of pathway inhibition. NA = value not available.

A canonical pathway enrichment analysis was performed using Ingenuity Pathway Analysis on all significantly changed genes with an absolute log_2_ fold change > 0.5. This analysis was complemented by an upstream regulator analysis. The results of the canonical pathway enrichment analysis are listed in Figure 5, and depict the significantly enriched pathways that were found in at least two treatments and in two doses (indicated by the −log_10_(*p*-value) > 1.3). Moreover, the z-score indicates whether the respective pathway was predicted to be activated or inhibited. As can be seen in Figure 5, exposure to CuO NPs induced the highest number of enriched pathways in BEAS-2B cells, followed by exposure to CuCl_2_ in HepG2 cells. The analyzed canonical pathways were categorized, and the three most abundant categories were immune response, the cellular (oxidative) stress response, and cytoskeleton signaling. Interestingly, the only pathway that was significantly enriched with a −log10(*p*-value) > 5 in all treatments was the NFE2L2 pathway. *NFE2L2* encodes the transcription factor NRF2. The only pathway that was predicted to be activated by all three treatments was metallothionein signaling (see Supplementary Materials, Figure S1, for a detailed heatmap). The SPINK1 cancer pathway was the only one consistently predicted to be inhibited and is involved in inflammatory signaling [24]. Another pathway shared by all three treatments was the Zn homeostasis signaling. While CuCl_2_ led to an activation of this pathway in both cell lines, exposure to CuO NPs in BEAS-2B cells resulted in an inhibition. Regarding CuO NP exposure in BEAS-2B cells, another significantly enriched pathway was cell cycle control. Additionally, a strong induction of stress signaling was observed, and signaling pathways involved in protein folding and ferroptosis were enriched and predicted to be activated in addition to NRF2.

**Figure 6:**
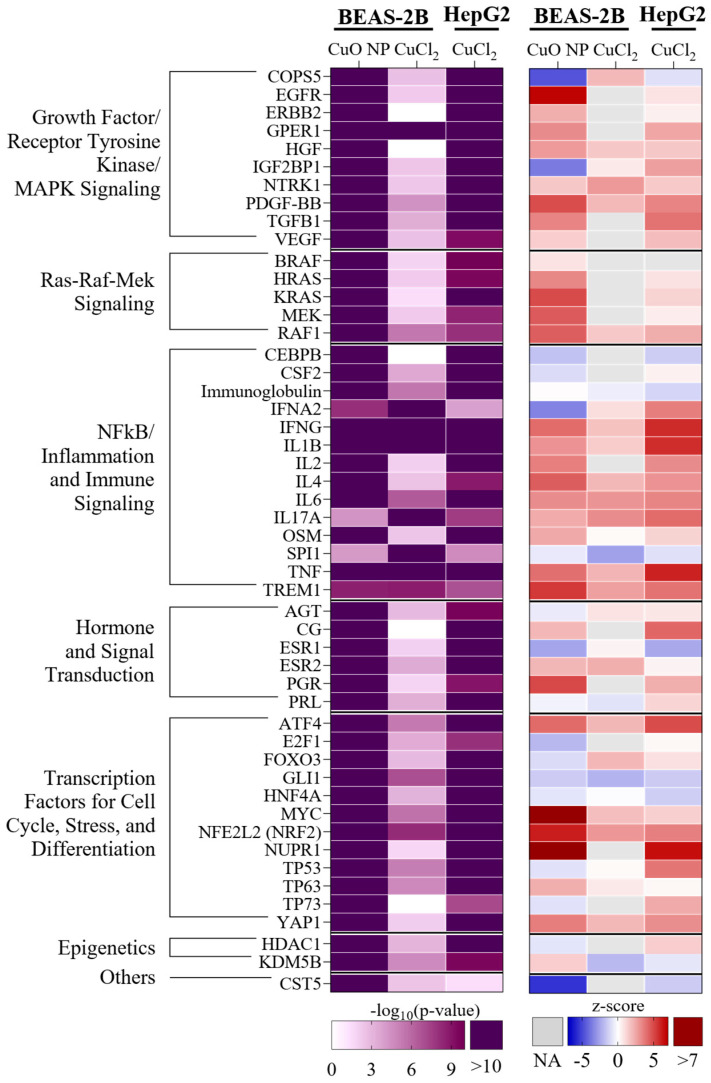
Upstream regulator analysis regarding the highest exposure concentration of 40 µg/mL Cu in BEAS-2B and HepG2 cells after 24 h exposure. The table shows all upstream regulators that were determined by ranking the sum of the −log_10_(*p*-values), assigned to different signaling categories. Significantly changed upstream regulators are depicted by a −log_10_(*p*-value) ≥ 1.3 (purple). The z-score provides information on whether a regulator was predicted to be induced (red), or inhibited (blue). NA = value not available.

We performed an analysis of the main 50 upstream regulators, which we determined by ranking the sum of −log_10_(*p*-values) (see Figure 6). The upstream regulators were categorized into signaling groups and assigned to growth factor signaling, the Ras-Raf-Mek signaling pathway, inflammation, hormone signaling, transcription factors involved in cell cycle control and stress response, and epigenetic signaling. Regarding the comparatively low intracellular Cu dose obtained by exposing BEAS-2B cells to CuCl_2_, changes in growth factor signaling and the stress response, and particularly inflammation, stood out. Under these treatment conditions, the strongest upregulation, as indicated by the z-score, was observed for interleukin (IL) *IL17A*, and the strongest downregulation was predicted for the upstream regulator *spi-1 proto-oncogene* (*SPI1*). Both *IL17A* and *SPI1* are related to inflammatory processes [25].

In BEAS-2B cells exposed to CuO NPs, the strongest induced upstream regulator with respect to the z-score was *nuclear protein 1* (*NUPR1*), followed by *NRF2*. Both *NUPR1* and *NRF2* are involved in the cellular oxidative stress response [26,27]. The most downregulated gene was *cystatin D* (*CST5*). In HepG2 cells exposed to CuCl_2_, the most activated upstream regulator was *tumor necrosis factor* (*TNF*), which is also involved in inflammation. The strongest inhibition was observed for *estrogen receptor 1* (*ESR1*). Overall, most of the upstream regulators belonged to the inflammation category, followed by transcription factors related to the cell cycle and the stress response. Taken together, exposure to CuCl_2_ in both cell lines seemed to have the most pronounced effect on inflammation. However, it is important to note that only 33 DEGs were found in BEAS-2B cells upon exposure to CuCl_2_. CuO NPs, which induced many more DEGs (4501), rather impacted the cellular stress signaling.

Notably, an additional gene set enrichment analysis using the KEGG pathway database confirmed that the only shared pathway among all treatments was MT signaling (see Supplementary Materials, S3). This analysis also revealed that CuO NPs induced the highest number of enriched pathways in BEAS-2B cells, with the cellular stress response representing the most affected category. Additionally, an enrichment in pathways related to translation was observed (see Supplementary Materials, Table S2).

Consequently, the NRF2 signaling pathway, the potential generation of ROS, cell cycle regulation, and cytokine release were examined.

#### NRF2 Signaling Pathway Analysis

**Figure 7:**
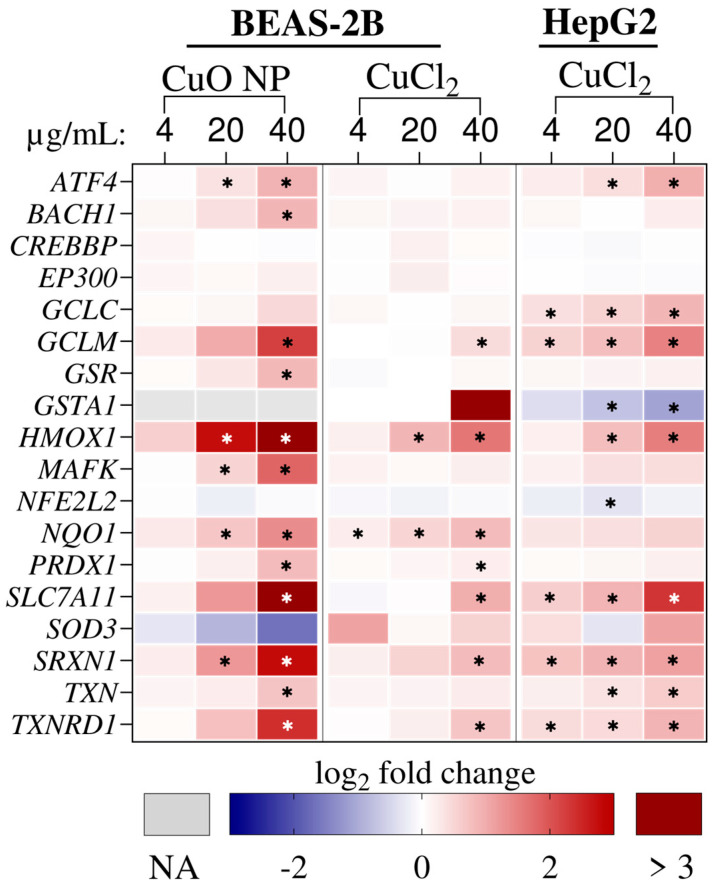
Genes found by canonical pathway enrichment analysis in the ‘NFE2L2 regulating antioxidant/detoxification enzymes’ network. Values are depicted as log_2_ fold change in three independent experiments compared to untreated controls. NA = value not available. *: adjusted *p*-value < 0.05.

Interestingly, the NRF2 signaling pathway was the only pathway that was predicted to be induced by all treatments with a −log_10_(*p*-value) > 5. CuO NP exposure in BEAS-2B cells and CuCl_2_ exposure in HepG2 cells resulted in NRF2 signaling activation, as indicated by the positive z-score. Figure 7 shows all genes from the network “NFE2L2 regulating antioxidant/detoxification enzymes”, which was obtained from canonical pathway enrichment analysis.

A concentration-dependent increase in expression was observed for most genes over all treatment conditions. Among the genes induced or suppressed by all three treatments, *HMOX1* exhibited the strongest induction. BEAS-2B cells exposed to CuO NPs showed 6.8-fold induction at 20 μg/mL Cu, and 50.6-fold induction at 40 μg/mL Cu. Moreover, *glutathione S-transferase alpha 1* (*GSTA1*) was non-significantly induced by Cu^2+^ exposure in BEAS-2B cells, reaching expression levels 26-fold higher than those of untreated controls. In contrast, *GSTA1* expression was significantly repressed to 47% in HepG2 cells exposed to Cu^2+^. Another difference was seen with *SOD3*, which decreased concentration-dependently to 30.9% in BEAS-2B cells exposed to CuO NPs. Other genes strongly induced by CuO NP exposure in lung cells were *glutamate-cysteine ligase modifier subunit* (*GCLM*), *SLC7A11*, *sulfiredoxin 1* (*SRXN1*), and *thioredoxin reductase 1* (*TXNRD1*). *SCL7A11* was also strongly induced by Cu^2+^ in HepG2 cells. The actual log_2_ fold change values can be found in the Supplementary Materials (Table S5). Overall, the data showed that NRF2-dependent antioxidant enzymes were significantly impacted by Cu, regardless of the compound or cell line studied. In this context, CuO NPs led to the most pronounced activation.

### 3.4. Oxidative Stress—ROS Generation

#### 3.4.1. Acellular ROS Potential of CuO NPs

Exposure to CuO NPs in BEAS-2B cells clearly demonstrated significant induction of oxidative stress signaling. Mechanistically, Cu(I) participates in Fenton-type reactions, generating hydroxyl radicals [6]. An increase in ROS challenges the cellular antioxidant response, which could explain the observed effects at the transcriptome level. Thus, we first examined whether CuO NPs lead to an increased oxidative stress response due to ROS bound to these particles intrinsically, by applying an acellular DCFH-DA assay.

**Figure 8:**
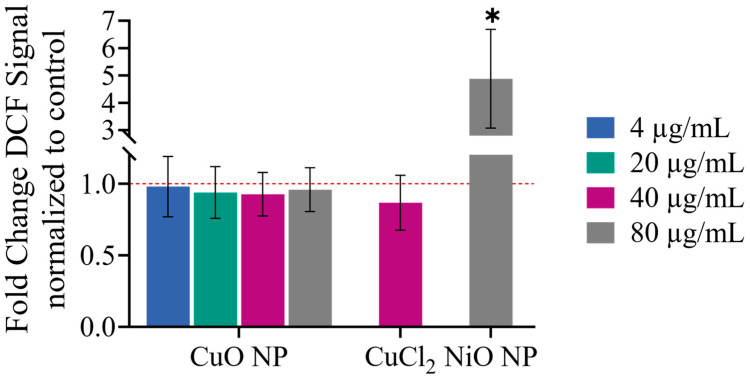
Acellular DCFH-DA assay examining the potential emergence of ROS that are bound to CuO NPs. CuCl_2_ as ion control was included at the highest concentration studied, and 80 µg/mL NiO NPs served as a positive control. The red dashed line at 1.0 represents a lack of change. An ordinary two-way ANOVA followed by Dunnett’s multiple comparison test of each treatment compared to untreated controls was applied: * *p* ≤ 0.05.

No increase in ROS was observed for any concentration of CuO NPs applied (see Figure 8). Therefore, the CuO NPs used in this study did not exhibit any intrinsic oxidative potential.

#### 3.4.2. Cellular ROS Generation by CuO NPs

**Figure 9:**
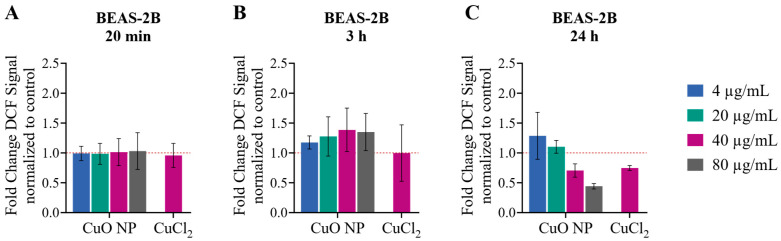
BEAS-2B cells were exposed to CuO NPs and CuCl_2_ for 20 min (**A**), 3 h (**B**), and 24 h (**C**). Induction of ROS was measured by the cellular DCFH-DA assay. Results are depicted as the mean of three independent experiments ± sem. The red dashed line at 1.0 represents a lack of change. An ordinary two-way ANOVA was applied, followed by Dunnett’s multiple comparison test. No exposure resulted in a significant change in DCF signal. The DCF signal is depicted as fold change normalized to untreated controls on the *y*-axis.

In addition to having an acellular ROS potential, CuO NPs can generate ROS intracellularly. For example, they can release Cu(I) ions that participate in Fenton-type reactions [6]. Therefore, a cellular ROS assay was performed. ROS are highly reactive molecules with an extremely short half-life [28]. Thus, this experiment included earlier timepoints than 24 h, namely 20 min and 3 h. To detect concentration-dependent effects, the experiment also examined a higher concentration of CuO NPs (80 µg/mL Cu). Figure 9 shows that CuO NPs did not induce an increase in ROS at any time point. Interestingly, exposure to Cu for 24 h resulted in a concentration-dependent decrease in the DCF signal. This could be due to interaction between the particles and the cellular membrane, which disturbs the uptake of DCFH into cells. This is particularly likely given that the cells were cultivated without FBS [10]. Furthermore, exposure to 80 µg/mL Cu for 24 h significantly reduced cell viability, which could contribute to the decreased signal. The positive control, H_2_O_2_, demonstrated assay functionality (see Supplementary Materials, Figure S3). Overall, it was concluded that, even though this assay did not detect an increase in ROS at the given time points, oxidative stress must have still occurred, as indicated by the transcriptome analysis. Therefore, compared to the transcriptome, the DCFH-DA assay is regarded as a less sensitive method for detecting an increase in oxidative stress.

### 3.5. Cell Cycle Distribution

**Figure 10:**
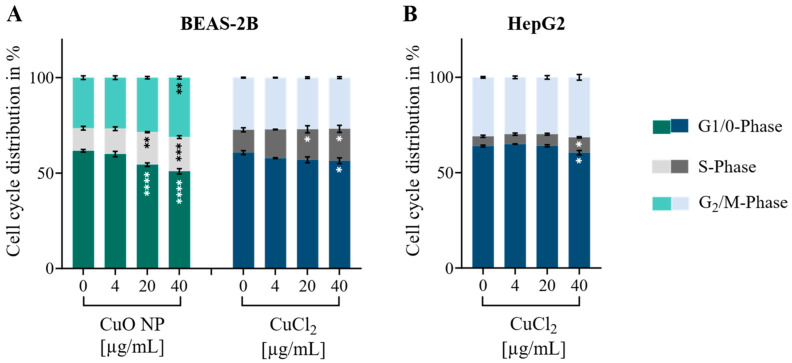
BEAS-2B cells (**A**) were exposed to CuO NPs and CuCl_2_, and HepG2 cells (**B**) were exposed to CuCl_2_ for 24 h. Cell cycle distribution was measured by flow cytometry. Bar charts depict the mean of three independent experiments ± sem. An ordinary two-way ANOVA followed by Dunnett’s multiple comparison test was applied: * *p* ≤ 0.05, ** *p* ≤ 0.01, *** *p* ≤ 0.001, **** *p* ≤ 0.0001.

Since significant alterations were found in both the canonical “Mitotic G_2_-G_2_/M phases” pathway and upstream regulators of cell cycle regulation, the impact of Cu on cell cycle phases on a functional level was examined by flow cytometry. BEAS-2B cells exposed to CuO NPs exhibited a concentration-dependent increase in the G_2_/M- and S-phases, while the G_0_/G_1_-phase decreased simultaneously (see Figure 10). Significant effects were observed at a non-cytotoxic exposure dose of 20 µg/mL. At the highest exposure concentration of 40 µg/mL Cu, the G_2_/M phase significantly increased from 26.5% to 31.1%, the S-phase increased from 11.8% to 17.9%, and the G_0_/G_1_-phase significantly decreased from 61.7% to 50.9%. A similar, albeit less pronounced, effect was observed in BEAS-2B cells exposed to Cu^2+^. In this case, the percentage of cells in the S-phase increased, while the percentage of cells in the G_0_/G_1_-phase decreased in a concentration-dependent manner. Interestingly, Cu^2+^ had the weakest effect on cell cycle regulation when exposed to HepG2 cells, even though intracellular Cu levels were higher than in BEAS-2B cells exposed to Cu ions. Only the highest exposure concentration of 40 µg/mL Cu resulted in a slight, but significant increase in the S-phase and a simultaneous decrease in the G_0_/G_1_-phase. Overall, only exposures to CuO NPs in BEAS-2B cells significantly impacted the G_2_/M-phase. This is consistent with transcriptome data showing that only CuO NP exposure led to an enrichment of G_2_/M signaling. In conclusion, Cu significantly impacted cell cycle regulation, even though non- or low-cytotoxic concentrations were used. Since the impact was observable independently of the treatment or cell line, it appears to be a general effect of Cu. Additionally, BEAS-2B cells were shown to be more susceptible than HepG2 cells to perturbations in cell cycle regulation on a functional level.

### 3.6. Inflammation—Cytokine Release

Regarding the most significantly enriched pathways upon Cu exposure at the transcriptome level, three pathways contained interferon signaling. Moreover, the upstream regulator analysis revealed 14 inflammatory cytokines, representing the most abundant category. Thus, inflammation was analyzed further on a functional level, and cytokine release was studied using a multiplex approach.

**Figure 11:**
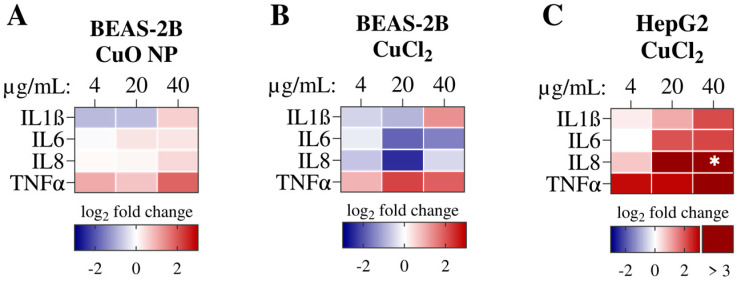
BEAS-2B cells were exposed to CuO NPs (**A**) and CuCl_2_ (**B**), and HepG2 cells were exposed to CuCl_2_ (**C**) for 24 h. Cytokine release was determined by ELISA-based multiplex assays. The heatmaps represent the mean of three independent experiments, depicted as log_2_ fold change. An ordinary two-way ANOVA followed by Dunnett’s multiple comparison test was applied: * *p* ≤ 0.05.

The heatmaps in Figure 11 depict mean cytokine release values for the proinflammatory cytokines IL1ß, IL6, IL8, and TNFα. BEAS-2B cells exposed to CuO NPs at the highest exposure dose exhibited a non-significant increase in all four cytokines. While the effect was less pronounced than in the transcriptome data, it was consistent with the z-score of the upstream regulator analysis observed in RNA sequencing analysis. Consistent with the transcriptome data, HepG2 cells exposed to Cu ions exhibited the strongest increase in IL1ß, IL6, and TNFα release. IL8 release was significantly increased by 33.5-fold compared to untreated controls. Therefore, the inflammatory response was most pronounced in HepG2 cells exposed to Cu. Cytokine release data from BEAS-2B cells exposed to CuCl_2_ produced partly contradictory results compared to the transcriptome data. Although CuCl_2_ led to the activation of proinflammatory cytokines, such as *IL6*, on the transcriptomic level, this assay observed a reduction in the release of IL6 and IL8. This discrepancy may be due to the small number of DEGs found in BEAS-2B cells exposed to CuCl_2_, and the significance of the upstream regulator analysis of *IL6* is limited as well.

## 4. Discussion

In this study, we compared the cellular responses to Cu overload in two organ-relevant cell models: human hepatoblastoma (HepG2) liver cells and human bronchial epithelial lung (BEAS-2B) cells. We applied transcriptomic profiling and included studies on various functional endpoints, such as uptake, cell cycle regulation, and cytokine release, with a focus on dose-dependent and cell-type-dependent effects.

Overall, this study emphasizes the pivotal role of transcriptomic profiling in unveiling cell-type-specific responses to Cu exposure, with functional-level investigations offering supplementary insights. Although global effects on the transcriptome level were consistently associated with respective cellular uptake levels of Cu, distinct differences were observed. Regarding cellular uptake, exposure to CuO NPs was shown to result in a much higher cellular Cu load than exposure to Cu ions. This is likely the main reason for their increased toxicity. Mechanistically, it has been reported that the increased uptake occurs via a Trojan-horse type mechanism in lung cells [29,30]. Thus, NPs are taken up via clathrin-dependent endocytosis. Following the fusion of endosomes with lysosomes and following acidification, Cu ions are released from the highly soluble CuO NPs, resulting in a massive intracellular Cu ion overload [19,30,31]. Furthermore, the increased uptake of Cu ions in HepG2 cells compared to BEAS-2B cells may be due to hepatocyte characteristics. Hepatocytes play a major role in physiological Cu storage and are equipped with highly efficient Cu transporters [32]. Within the present study, CuO NPs were not studied in HepG2 cells, since the liver is not expected to be considerably exposed to the NPs in vivo, neither after inhalation nor after oral intake, due to their high solubility in acidic environments, as present in lung lysosomes or in the stomach. Different studies have demonstrated rapid and extensive dissolution of CuO NPs in biological media [33], reaching almost complete solubilization in artificial lysosomal fluid within 2 h [34]. Furthermore, it was shown that the solubility of the same CuO NPs used also in this study reached 97.3% in a dynamic dissolution model conducted with a phagolysosomal simulant fluid (pH 4.5), representative of uptake via the lung upon endocytosis [19]. These findings support that the acidic conditions drive CuO dissolution and the subsequent release of soluble Cu species. Similarly, pronounced dissolution of CuO NPs has been demonstrated under gastric conditions, meaning that CuO NPs are largely converted to soluble Cu ions before intestinal uptake [11]. Even in the case of rodent studies on largely insoluble iridium NPs or gold NPs, after inhalation only a small amount of approximately 0.2% was reported to translocate to secondary organs, such as the liver [35,36]. Consequently, in liver cells, Cu ion exposure was considered relevant also for highly soluble CuO NPs.

In this context, it is important to note that the sub-cytotoxic exposure concentrations chosen for the experiments in this study are relevant to human exposure and physiological levels. Cu burden in the liver of healthy individuals can reach up to 55 µg/g of the liver dry weight [5]. Assuming a water content of 70%, this equates to 16.5 µg/mL Cu. Thus, the medium exposure concentration used in this study reflects a Cu content relevant to a healthy human body. In patients diagnosed with Wilson disease, minimum Cu levels of 250 µg/g in liver dry weight can be found, equivalent to 75 µg/mL Cu [5]. Consequently, the applied concentrations covered normal to high Cu loads in the liver, which may be relevant for healthy individuals. The Cu burden in the lungs can vary greatly. Using MPPD modeling for different NPs not containing Cu, researchers demonstrated that alveolar retention after NP exposure over 24 h resulted in approximately 0.57 µg/mL [37]. However, the buildup of NPs in so-called hot spots in the lungs, i.e., airway bifurcations, can result in more than two orders of magnitude higher particle doses when compared to average deposition [38]. Thus, the lowest exposure concentration is particularly relevant, while the highest exposure concentration further reflects potentially high exposure concentrations found in airway bifurcations, especially on conditions of particle overload.

Through transcriptome analysis, we demonstrated that, regardless of the Cu compound or cell line, MTs appear to be the most sensitive marker of an increase in intracellular Cu. MTs are metalloproteins with 20 cysteine residues that bind metal ions with high affinity. Intracellular metal ions, such as Zn, Fe, or Cu, can mechanistically bind to the Zn-finger domains of the Metal Regulatory Transcription Factor 1 (MTF-1), thereby activating it. MTF1 then translocates to the nucleus and drives the expression of genes such as MTs [39]. Interestingly, changes in gene expression at the lowest exposure dose were almost exclusively restricted to MT induction. An increase in MTs is not yet a cellular danger signal, but rather an adaptive cellular mechanism involved in metal homeostasis that prevents elevated levels of “labile”, i.e., loosely bound, metal ions. Thus, no toxic effect of Cu was observed upon exposure to 4 µg/mL Cu, indicating that a toxicity threshold lies above this level. This observation is consistent with previous reports on A549 and HepG2 cells exposed to Cu compounds and ions, respectively [13,15]. When comparing intracellular Cu levels upon exposure to equivalent concentrations, exposure of BEAS-2B cells to Cu^2+^ resulted in the lowest intracellular Cu load. Notably, even with this exposure, the MT expression increased markedly in a concentration-dependent manner. A massive Cu overload, as induced by CuO NP exposure, could overwhelm this protective mechanism, and exceed the capacity of MTs [40]. This would explain why MT induction was observed at all concentrations and by all treatments at the transcriptome level. In addition to interacting with Zn-binding structures, Cu also interacts with the Fe homeostasis. This was indicated by significant induction of Zn- and Fe homeostasis signaling in the canonical pathway enrichment analysis. In this context, Song et al. also suggested that there are shared characteristics in the transcriptome effects upon exposure to Cu^2+^ compared to Zn^2+^ in HepG2 cells [15]. Furthermore, CuO NPs significantly induced the Fe-dependent programmed cell death mechanism, ferroptosis. Ferroptosis is characterized by a disturbance of SLC7A11, a cystine membrane transporter. This results in cellular GSH depletion, lipid peroxidation, decreased GPX4 activity, and increased cellular labile Fe [41,42]. The massive upregulation of *HMOX1*, which encodes the respective protein HO-1, could have increased the labile Fe pool. HO-1 degrades heme molecules and thereby liberates free Fe ions [43]. Thus, an increase in labile Fe contributes to increased oxidative stress and further challenges the limited metal-binding capacity of MTs. The induction of ferroptosis by Cu has previously been described in kidney cells, for example [44]. Furthermore, our study reveals a mechanistic link between these oxidative stress pathways because both *HMOX1* and *SLC7A11* expression are associated with NRF2 signaling. Figure 7 shows a concentration-dependent increase in both *HMOX1* and *SLC7A11* with all treatments. CuO NP exposure in BEAS-2B cells induced the most pronounced effect. Notably, glutamate cysteine ligase subunits *GCLC* and *GCLM* were also induced in a concentration-dependent manner. This enzyme is relevant for GSH synthesis [45]. Thus, NRF2 and ferroptosis signaling are connected. Previous studies have shown that the NRF2 pathway can be induced by ferroptosis-related stress signaling [42]. From a mechanistic perspective, the induction of NRF2 signaling, including increased *SLC7A11* expression, could represent a cellular response not only to oxidative stress and increased intracellular Cu, but also to ferroptosis. Increasing *SLC7A11* gene expression induces the transport protein, allowing more cystine to enter cells. Once inside the cell, cystine is converted to cysteine, which modulates GSH synthesis [46]. In addition to increases in *GCLC*, *GCLM*, and *SCL7A11*, the concentration-dependent decrease in *GSTA1* to 47% in HepG2 further indicates changes in the GSH metabolism. In this context, researchers previously suggested that HO-1 induction results in a GSH depletion in HepG2 cells [47], which could explain the observed reduction in *GSTA1*. Also, in support of this assumption, a concentration-dependent decrease in the GSH/GSSG ratio in HepG2 cells exposed to CuCl_2_ concentrations comparable to those used in this study was described by Sasula et al. [48]. Interestingly, *GSTA1* was markedly, though not significantly, induced by Cu^2+^ in BEAS-2B cells, indicating cell-type-specific differences in cellular responses to Cu. While exposure to Cu may have overwhelmed the detoxification mechanism in liver cells, lung cells still had a robust antioxidant response. Given the different intracellular Cu loads, these results suggest that certain thresholds may exist that determine cellular fate in the event of moderate or severe Cu overload. Overall, the induction of oxidative stress signaling was considered a driving mechanism of Cu-induced toxicity, whereby the NRF2 pathway seems to be a highly susceptible target. Besides differences in predicted NRF2 activation, which could be based on the intracellular Cu load, this pathway was significantly impacted by Cu, independent of Cu compound or cell type. Under physiological conditions, the transcription factor NRF2 is bound to its inhibitor, kelch-like ECH-associated protein 1 (KEAP1), in the cytosol [49]. An increase in oxidative stress causes the cysteine residues of KEAP1 to oxidize, leading to a conformational change in the protein and the subsequent release of NRF2. NRF2 can then translocate to the nucleus and activate the gene expression of genes, including antioxidant genes [50]. Therefore, NRF2 is considered a main cellular regulator of the antioxidant defense system [51]. In another transcriptome study of HepG2 cells exposed to Cu, metal-binding and oxidative stress pathways were found to be the most enriched [16]. In general, the induction of the NRF2 pathway appears to be a common toxicity mechanism of Cu [52,53]. Song et al. also observed a pronounced induction of the cellular stress response as well as NRF2 activation in HepG2 cells exposed to Cu^2+^ [15,16].

Basically, oxidative stress is believed to be induced by an increase in ROS. However, no increase in ROS driven by CuO NPs was observed at the functional level. This leads us to assume that the stress response signaling may also be based on other mechanistic factors, such as the targeted modulation of the cellular redox regulation by Cu. Therefore, the specific impact of Cu on redox-regulated proteins, such as KEAP1, should be studied in the future. The results obtained by the DCFH-DA assay could be due to its limitations. For example, not all types of ROS can be detected by this assay, and it may not detect changes in mitochondrial ROS or have limitations in its reaction time [28]. In contrast, Sasula et al. demonstrated a significant increase in the levels of cellular H_2_O_2_ in HepG2 cells exposed to CuCl_2_ after 24 h [48]. In our study, an induction of the antioxidant defense by NRF2 signaling could efficiently reduce ROS levels in the cells. This could also explain the concentration-dependent decrease in the DCF signal after 24 h of CuO NP exposure in lung cells, as the NRF2 pathway-related genes were induced in a concentration-dependent manner.

In this study, another effect observed was the significant impact on MAPK signaling, which includes upstream regulators such as Ras-Raf-Mek. MAPK signaling can be initiated by EGFR activation, for example, and regulates proliferation and cell survival [54]. EGFR activation by CuO NPs was predicted in BEAS-2B cells, reaching a z-score of approximately 7 at the highest exposure concentration, and exhibiting a log_2_ fold change of 0.46 (raw data can be found in ArrayExpress, accession number E-MTAB-16794). In this context, Piret et al. hypothesized that, in addition to redox-sensitive transcription factors, MAPK signaling could be a main driver of CuO NP toxicity [55]. Various other studies have also consistently observed activation of MAPK signaling [13,15]. Since this pathway drives cell cycle progression and other cascades, and since a significant induction of the G_2_/M-phase pathway was only observed in BEAS-2B cells exposed to CuO NPs, the regulation of the cell cycle was studied further. We showed that the data obtained by flow cytometry were consistent with transcriptomic results of CuO NP exposure. Indeed, a G_2_/M-phase arrest was observed at the highest concentration. However, exposure to Cu ions did not lead to changes in the G_2_/M-phase in either BEAS-2B or HepG2 cells. This indicates that severe Cu overload induces a G_2_/M-arrest, strengthening the hypothesis of an intracellular Cu threshold that results in a switch in cellular signaling. Moreover, cell cycle analysis further revealed a concentration-dependent impact on the G_0_/G_1_- and S-phases with all treatments. While the number of cells in the S-phase increased significantly already at noncytotoxic exposure doses, the number of cells in the G_0_/G_1_-phase decreased. An increase in cells in the S-phase may reflect an extension of DNA repair through an increase in DNA replication over time. This indicates that potentially occurring DNA damage was manageable for the cells and that apoptosis or necrosis was not induced [56]. This is consistent with the fact that only non- or low-cytotoxic concentrations of Cu were applied. Furthermore, also a transcriptome study in A549 cells exposed to both CuO NPs and Cu ions reported a substantial effect on a G_1_- and G_2_-phase cell cycle arrest [13]. Interestingly, despite the higher intracellular Cu load in HepG2 cells compared to BEAS-2B cells, and similar CuCl_2_ cytotoxicity, the effects on cell cycle regulation were much less pronounced. Therefore, our data provide important information on how susceptible different organ-relevant cell types are to Cu overload.

Ultimately, the strongest predicted activation effect of Cu was observed with respect to NFkB signaling and, consequently, inflammation. A consistently induced upstream regulator across all treatments was *IL17A*. An in silico analysis of both human and murine data revealed increased IL17 transcripts by several NPs [57]. Hence, NFkB-mediated signaling was considered another main target of acute Cu toxicity. While other researchers also have observed this effect in HepG2 cells exposed to Cu^2+^ [15,16], Hanagata et al. did not report inflammation as a primary effect in A549 cells exposed to the respective Cu compounds [13]. This suggests that different cell types exhibit distinct cellular responses to Cu exposure.

Of note, Piret and co-authors thoroughly studied the cellular responses of HepG2 cells exposed to different CuO NPs, including transcriptomic profiling. The authors reported that both the oxidative stress response and inflammation were initiated, and that an increase in ROS was measured. They also reported the activation of all three signaling pathways discussed in this study: NRF2, NFkB, and MAPK signaling [55]. These findings suggest that in liver cells, the cellular responses to Cu exposure are likely compound-independent, indicating that Cu ions may be the primary cause of toxicity.

Interestingly, a study investigating cellular responses to welding particles revealed that lung inflammation is driven by oxidative stress and the induction of NRF2 signaling [58]. In this context, our study provides useful data on the connection between NFkB and NRF2 signaling. Consequently, we examined cytokine release. Regarding HepG2 cells, the predicted activation of NFkB-related upstream regulators, such as TNF, was also observed through cytokine release. Thus, the activation of NFkB signaling predicted by transcriptome analysis was confirmed at the functional level, indicating the substantial impact of Cu on NFkB signaling. Interestingly, while Sasula et al. also found increased expression of both inflammatory gene markers and cytokine levels in HepG2 cells exposed to Cu ions, this could not be attributed to a substantial effect of Cu on NFkB promoter activity [48]. This again could suggest a closer link between oxidative stress signaling and inflammation. Furthermore, while the activation of proinflammatory cytokines predicted by Cu ions in HepG2 cells reflected the transcriptome analysis results in our study, this could not be applied to lung cells. In this case, CuO NP exposure led only to a slight, non-significant increase in proinflammatory cytokine release, which was restricted to the highest exposure dose. Regarding IL6 and IL8 release from BEAS-2B cells exposed to CuCl_2_, a trend toward decline compared to untreated controls was observed. This again highlights cell-type-specific differences in the relationship between transcriptional regulation and cellular fate. The reduced release of IL6 and IL8 may reflect a protective adaptation in lung cells, whereby intracellular signaling remains active, but cytokine secretion is attenuated to limit excessive cell damage. In contrast, this regulatory mechanism was not evident in liver cells, possibly due to their substantially higher intracellular Cu burden. Under these conditions, an increased cytokine release may serve as an important danger signal to surrounding tissue structures in vivo. However, this could also be a cell-type-specific difference that should be examined further in future studies.

## 5. Conclusions

In conclusion, it was demonstrated that Cu compounds can induce multiple changes in signaling, particularly in the NFkB, MAPK, and NRF2 signaling pathways. Concentration plays an important role; the lowest exposure dose applied in this study did not result in toxic effects. Additionally, exposure to specific Cu compounds can result in significant differences in cellular effects. Transcriptome profiling is a highly sensitive method for studying Cu toxicity. However, different organ-relevant cell models must be included to gain appropriate mechanistic insights and distinguish effects based on cellular susceptibility. Finally, integrating toxicity studies at the functional level with transcriptome profiling is a powerful way to improve our mechanistic understanding of metal toxicity.

## Figures and Tables

**Table 1:** Exposure concentrations comparing different units regarding exposures to CuO NPs and Cu ions.

Total Cu Concentration in µg/mL	Concentration of CuO NPs in µg/mL	Cu Concentration in µg/cm^2^	Cu Concentration in µM
4	5	~0.8	63
20	25	~4.0	314
40	50	~8.0	628

**Table 2:** Number of differentially expressed genes (DEGs) in BEAS-2B cells treated with CuO NPs and CuCl_2_ as well as HepG2 cells treated with CuCl_2_, compared to untreated controls. All treatments included exposure concentrations of 4–40 µg/mL Cu (stated by low, medium and high dose). Significant changes in gene expression were considered all FDR-corrected *p*-values ≤ 0.5. For IPA analysis, only DEGs with −0.5 > log_2_ fold change > 0.5 were used, indicated by the number of mapped DEGs.

	BEAS-2B Cells				HepG2 Cells	
	CuO NPs		CuCl_2_		CuCl_2_	
Dose	Total Nr. of DEGs	Mapped DEGs	Total Nr. of DEGs	Mapped DEGs	Total Nr. of DEGs	Mapped DEGs
Low	10	10	4	4	6	6
Medium	1984	1538	14	13	46	39
High	4698	3832	33	30	520	375

## Data Availability

Raw data of RNA sequencing analysis is published in ArrayExpress, accession number E-MTAB-16794. Other data used in this study is available upon request from the corresponding author AH for any researcher of academic institutes who meet criteria to gain access to confidential data.

## Supplementary Materials

The following supporting information can be downloaded at: https://www.mdpi.com/article/10.3390/nano16100590/s1. Table S1. Canonical pathways enrichment analysis regarding BEAS-2B and HepG2 cells exposed to 40 µg/mL Cu for 24 h. The table shows the top 40 canonical pathways that were predicted to be enriched related to the sum of all *p*-values from all conditions. The level of significance is represented by the FDR-corrected −log_10_(*p*-value), whereby any value greater than 1.3 is considered significant, depicted in purple. The Z-Score further provides information on pathway activation, by positive values (red) or predicted inhibition by negative values (blue). Table S2. Data obtained on the Gene Set Enrichment Analysis of BEAS-2B cells exposed to CuO NP (40 µg/mL Cu). FDR = false discovery rate, ES = enrichment ratio, NES = normalized enriched ratio. Table S3. Data obtained on the Gene Set Enrichment Analysis of BEAS-2B cells exposed to CuCl_2_ (40 µg/mL Cu). FDR = false discovery rate, ES = enrichment ratio, NES = normalized enriched ratio. Table S4. Data obtained on the Gene Set Enrichment Analysis of HepG2 cells exposed to CuCl_2_ (40 µg/mL Cu). FDR = false discovery rate, ES = enrichment ratio, NES = normalized enriched ratio. Table S5. Genes in the NFE2L2 regulating anti-oxidant/detoxification enzymes network. Depicted as log_2_ fold change values of three independent experiments. Figure S1. Genes found by canonical pathway analysis in the ‘metallothioneins bind metals’ network. Figure S2. Enriched pathways determined by the normalized enrichment ratio through Gene Set Enrichment Analysis of KEGG Pathways with a FDR < 0.05. Figure S3. Positive controls (PCs) used for the cellular DCFH-DA assay. BEAS-2B cells were incubated with both compounds for 20 min. Bar chart represents mean of all independent experiments ± sem. An ordinary one-way ANOVA followed by Dunnett’s multiple comparison test was applied: ** *p* ≤ 0.01.

## Abbreviations

The following abbreviations are used in this manuscript:

CST5	cystatin D
Cu	copper
CuCl_2_	copper chloride
CuO	copper(II) oxide
DCFH-DA	2′,7′-dichlorodihydrofluorescein diacetate
DEGs	differentially expressed genes
DMEM	Dulbecco’s Modified Eagle Medium
EGR1	early growth response 1
ESR1	estrogen receptor 1
FBS	fetal bovine serum
Fe	iron
GCLC	glutamate cysteine ligase catalytic subunit
GCLM	glutamate-cysteine ligase modifier subunit
GF-AAS	graphite furnace atomic absorption spectroscopy
GSH	glutathione
GSTA1	glutathione S-transferase alpha 1
HMOX1	heme oxygenase 1
HSPA1A	heat shock protein family A member 1A
ID3	inhibitor of DNA binding 3
KEAP1	kelch-like ECH-associated protein 1
KGM	keratinocyte growth medium
LAL	limulus amebocyte lysate
MAFF	MAF bZIP transcription factor F
MAPK	mitogen-activated protein kinases
MTF-1	metal regulatory transcription factor 1
MTs	metallothioneins
NFE2L2/NRF2	nuclear factor erythroid 2-related factor 2
NFkB	nuclear factor kappa-light-chain-enhancer of activated B cells
NiO	nickel(II) oxide
NPS	nanoparticles
NUPR1	nuclear protein 1
OSGIN1	oxidative stress induced growth inhibitor 1
PBS	phosphate-buffered saline
PPP1R15A	protein phosphatase 1 regulatory subunit 15A
PSAT1	phosphoserine aminotransferase 1
ROS	reactive oxygen species
SEL1L3	SEL1L family member 3
sem	standard error of the mean
SLC30A11	transmembrane protein 163
SLC7A11	solute carrier family 7 member 11
SOD	superoxide dismutase
SQSTM1	sequestosome 1
SRXN1	sulfiredoxin 1
TNF	tumor necrosis factor
TXNRD1 Zn	thioredoxin reductase 1 zinc
