# Supplementary material for: Transcriptomic Profiling of Toxic Copper Overload Induced by CuO Nanoparticles or Copper Ions in Human Lung Epithelial and Liver Cells
Source: Nanomaterials (Basel). 2026 May 12;16(10):590. doi: 10.3390/nano16100590 (PMC13209870; doi:10.3390/nano16100590)
Supplement: Supplementary file 1 [file nanomaterials-16-00590-s001.zip › nanomaterials-4245016-supplementary.pdf]

nanomaterials MDPI

Supplementary Material

Transcriptomic profiling of toxic copper overload induced by CuO nanoparticles or copper ions in human lung epithelial and liver cells

Jana Kuhn[1], Anda Gliga[2], Cheyenne I. Aissouni[1], Anna Glowacki[2], Marlene Parsdorfer[1], Martin Link[1], Hanna L. Karlsson[3*], Andrea Hartwig[1*]

[1] Department of Food Chemistry and Toxicology, Institute of Applied Biosciences (IAB), Karlsruhe Institute of Technology, Karlsruhe, Germany
[2] Unit of Metals and Health, Institute of Environmental Medicine, Karolinska Institutet, Stockholm, Sweden
[3] Department of Pharmaceutical Biosciences, Uppsala Universitet, Uppsala, Sweden
* Correspondence: hanna.l.karlsson@uu.se (HK); andrea.hartwig@kit.edu (AH)

S1. Main Canonical Pathways

Table S1. Canonical pathways enrichment analysis regarding BEAS-2B and HepG2 cells exposed to 40 µg/mL Cu for 24 h. The table shows the top 40 canonical pathways that were predicted to be enriched related to the sum of all p-values from all conditions. The level of significance is represented by the FDR-corrected -log10(p-value), whereby any value greater than 1.3 is considered significant, depicted in purple. The Z-Score further provides information on pathway activation, by positive values (red) or predicted inhibition by negative values (blue).

	BEAS-2B Cells				HepG2 Cells	
	CuO NP		$CuCl_2$		$CuCl_2$	
**Enriched Canonical Pathways**	-log10 (p-value)	z-score	-log10 (p-value)	z-score	-log10 (p-value)	z-score
BAG2 Signaling Pathway	18,806	-0,243	3,934	N/A	1,517	N/A
Cachexia Signaling Pathway	11,017	2,269	2,119	N/A	4,267	1,5
Cell Cycle Checkpoints	16,758	0,211	0,000	N/A	5,231	-3,873
EIF2 Signaling	13,886	1,622	0,593	N/A	1,943	0,378
Ferroptosis Signaling Pathway	4,359	2,058	3,338	N/A	10,146	0,5
Hedgehog 'off' state	15,511	3,703	0,000	N/A	1,691	N/A
Hedgehog 'on' state	15,635	2,994	0,000	N/A	0,231	N/A
HIF1α Signaling	5,175	-0,146	4,093	N/A	5,146	1,155
Huntington's Disease Signaling	14,142	-0,392	2,418	N/A	0,257	N/A
Interferon alpha/beta signaling	2,700	-1,342	5,836	-1	0,000	N/A
Major pathway of rRNA processing in the nucleolus and cytosol	34,033	9,381	0,000	N/A	0,000	N/A
MAPK6/MAPK4 signaling	17,226	0,775	0,000	N/A	0,000	N/A
Metabolism of polyamines	16,694	5,916	1,200	N/A	0,714	N/A
Metallothioneins bind metals	2,834	2,449	15,314	2,449	12,743	2,828
Microautophagy Signaling Pathway	16,437	5,217	0,000	N/A	0,431	N/A

 https://doi.org/10.3390/xxxxx

Mitotic G1 phase and G1/S transition	21,605	4,464	0,000	N/A	1,452	2
Mitotic G2-G2/M phases	16,458	1,86	0,000	N/A	4,660	-0,905
Mitotic Metaphase and Anaphase	16,264	2,333	0,000	N/A	6,729	-2,324
NFE2L2 regulating anti-oxidant/detoxification enzymes	4,971	3,464	5,675	N/A	10,980	2,333
Nuclear Cytoskeleton Signaling Pathway	5,165	-1,414	0,663	N/A	6,352	-0,577
Protein Ubiquitination Pathway	14,582	5,513	3,625	N/A	0,479	-1,342
Pulmonary Fibrosis Idiopathic Signaling Pathway	10,299	-3,491	0,518	N/A	3,218	2,496
RAF/MAP kinase cascade	15,828	-0,272	0,000	N/A	1,158	-0,378
Regulation of mitotic cell cycle	16,544	3,618	0,000	N/A	0,922	N/A
Regulation of mRNA stability by proteins that bind AU-rich elements	17,135	6,41	1,028	N/A	0,000	N/A
Response of EIF2AK1 (HRI) to heme deficiency	4,489	3	1,785	N/A	9,306	2,646
RHO GTPase cycle	14,953	0,271	0,000	N/A	2,019	1,387
RHO GTPases Activate Formins	7,077	-0,309	0,000	N/A	10,003	-0,775
Role of PKR in Interferon Induction and Antiviral Response	6,806	-1	3,329	N/A	2,528	1,342
rRNA modification in the nucleus and cytosol	24,672	6,403	0,000	N/A	0,000	N/A
S Phase	21,171	3,606	0,000	N/A	0,000	N/A
Senescence Pathway	7,271	0	0,000	N/A	5,337	1,291
Signaling by ROBO receptors	21,169	3,96	0,000	N/A	0,000	N/A
Sirtuin Signaling Pathway	15,419	-1,432	1,371	N/A	4,760	-1,265
SPINK1 General Cancer Pathway	0,742	-2,887	9,998	-2,449	7,600	-1,897
Stress Granule Signaling Pathway	12,367	-1,342	3,692	-2	1,505	-0,707
TP53 Regulates Transcription of Cell Cycle Genes	8,648	-2,041	0,000	N/A	2,348	-1
tRNA Charging	8,886	4,359	0,000	N/A	7,412	2,828
Unfolded protein response	7,360	2,357	5,503	N/A	2,062	0,447
Zn Homeostasis Signaling Pathway	0,000	-1,212	4,772	2,828	0,954	2,524

S2. *Metallothionein Saturation*

Depicted in Figure S1 are all genes that were found for ‘metallothioneins bind metals’ network in the canonical pathways analysis. It shows that, independent of treatment and cell line, MTs were concentration-dependently induced. At the lowest exposure dose, *MT1G* was most induced by both Cu compounds in BEAS-2B cells, while *MT1B* was most induced by CuCl2 in HepG2 cells.

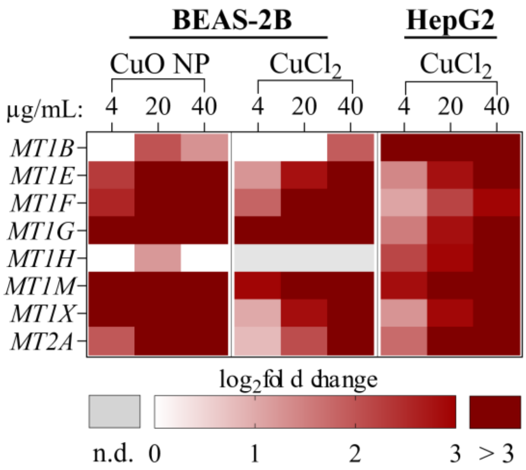

Figure S1. Genes found by canonical pathway analysis in the 'metallothioneins bind metals' network.

S3. *Gene Set Enrichment Analysis with KEGG Pathways*

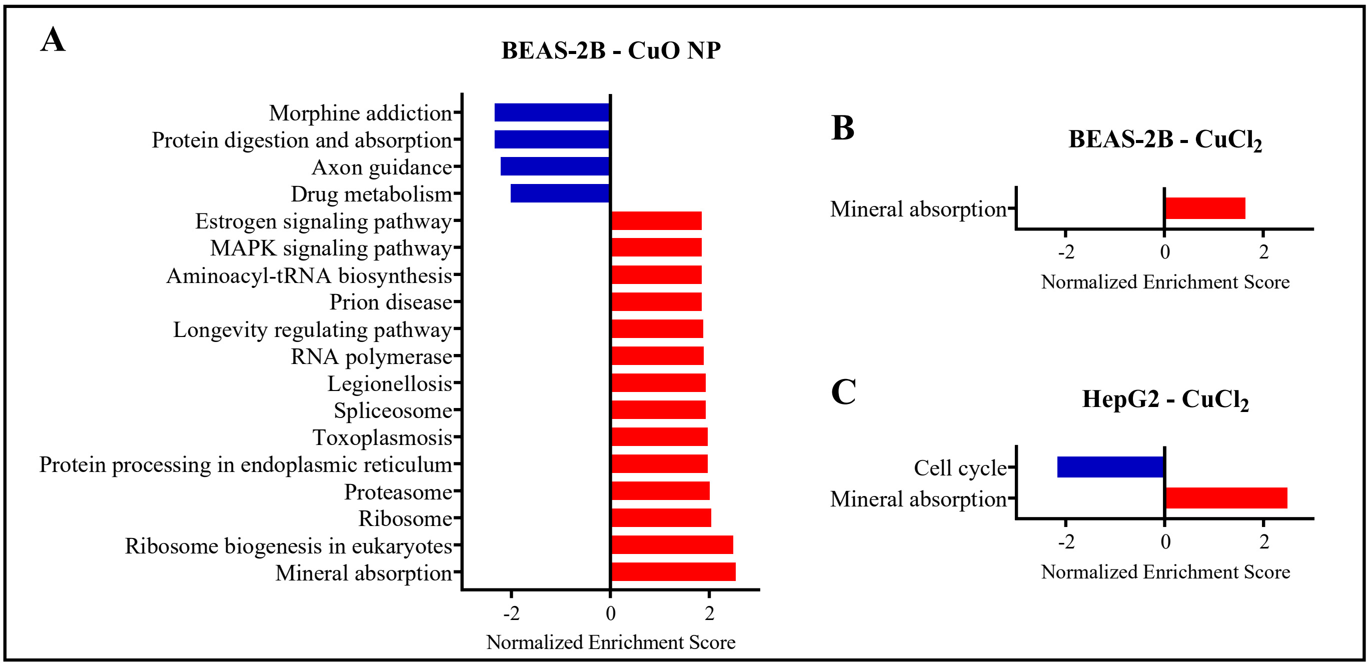

Figure S2. Enriched pathways determined by the normalized enrichment ratio through Gene Set Enrichment Analysis of KEGG Pathways with a FDR < 0.05.

Table S2. Data obtained on the Gene Set Enrichment Analysis of BEAS-2B cells exposed to CuO NP (40 µg/mL Cu). FDR = false discovery rate, ES = enrichment ratio, NES = normalized enriched ratio.

Assigned Category	Pathway Description	p-value	FDR	ES	NES	Nr. of Genes
Cellular Stress Response	Proteasome	<2.2e-16	0.024622	0.54891	2.0068	28
	Protein processing in endoplasmic reticulum	<2.2e-16	0.030932	0.47543	1.9700	25
	Toxoplasmosis	0.0033389	0.028213	0.54294	1.9616	12
	Spliceosome	0.0032787	0.036384	0.48865	1.9265	3
	Legionellosis	0.0017153	0.032894	0.55649	1.9234	9

	Longevity regulating pathway	<2.2e-16	0.042012	0.59762	1.8781	3
	Prion disease	0.0015674	0.048755	0.41446	1.8506	57
	Estrogen signaling pathway	<2.2e-16	0.039627	0.47913	1.8454	6
Metal Homeostasis	Mineral absorption	<2.2e-16	<2.2e-16	0.80143	2.5334	8
Translation	Ribosome biogenesis in eukaryotes	<2.2e-16	<2.2e-16	0.59702	2.4850	47
	Ribosome	<2.2e-16	0.021545	0.47980	2.0339	67
	RNA polymerase	0.0033956	0.040866	0.61777	1.8898	13
MAPK Signaling	MAPK signaling pathway	<2.2e-16	0.042201	0.41160	1.8484	23
Axonal Guidance Signaling	Axon guidance	<2.2e-16	0.0017530	-0.49859	-2.2179	26
Cytoskeleton	Protein digestion and absorption	<2.2e-16	<2.2e-16	-0.60357	-2.3390	17
Others	Aminoacyl-tRNA biosynthesis	0.0071301	0.045077	0.55226	1.8494	19
	Drug metabolism	<2.2e-16	0.037798	-0.70634	-2.0145	5
	Morphine addiction	<2.2e-16	<2.2e-16	-0.69418	-2.3392	8

Table S3. Data obtained on the Gene Set Enrichment Analysis of BEAS-2B cells exposed to $CuCl_2$ (40 µg/mL Cu). FDR = false discovery rate, ES = enrichment ratio, NES = normalized enriched ratio.

**Assigned Category**	**Pathway Description**	**p-value**	**FDR**	**ES**	**NES**	**Nr. of Genes**
Metal Homeostasis	Mineral absorption	<2.2e-16	<2.2e-16	0.89179	1.6305	8

Table S4. Data obtained on the Gene Set Enrichment Analysis of HepG2 cells exposed to $CuCl_2$ (40 µg/mL Cu). FDR = false discovery rate, ES = enrichment ratio, NES = normalized enriched ratio.

**Assigned Category**	**Pathway Description**	**p-value**	**FDR**	**ES**	**NES**	**Nr. of Genes**
Metal Homeostasis	Mineral absorption	<2.2e-16	<2.2e-16	0.89179	1.6305	8
Cell Cycle	Cell Cycle	<2.2e-16	0.028815	-0.66432	-2.1763	8

S4. NRF2 Signaling Pathways – log_2 fold change values

Table S5. Genes in the NFE2L2 regulating anti-oxidant/detoxification enzymes network. Depicted as log_2 fold change values of three independent experiments.

	**BEAS-2B Cells**						**HepG2 Cells**		
	CuO NP			$CuCl_2$			$CuCl_2$		
µg/mL Cu:	4	20	40	4	20	40	4	20	40
ATF4	0,229	0,394	0,970	0,025	0,349	0,928	0,128	0,015	0,156
BACH1	0,083	0,011	0,227	0,104	0,383	0,880	0,095	0,135	0,152

CREBBP	-0,046	-0,071	0,015	0,108	0,002	-0,035	0,021	0,163	0,051
EP300	-0,018	-0,058	-0,057	0,115	0,079	0,191	0,019	0,223	0,042
GCLC	0,381	0,538	0,876	0,048	0,104	0,469	0,089	-0,021	0,100
GCLM	0,553	0,765	1,494	0,250	1,004	2,223	-0,002	-0,027	0,430
GSR	0,100	0,136	0,161	0,052	0,301	0,839	-0,061	-0,010	0,085
GSTA1	-0,412	-0,732	-1,106	N/A	N/A	N/A	0,000	0,000	4,703
HMOX1	0,183	0,766	1,520	0,575	2,760	5,664	0,200	0,878	1,619
MAFK	0,162	0,385	0,415	0,012	0,514	1,801	0,158	0,071	0,210
NFE2L2	-0,214	-0,348	-0,170	-0,024	-0,206	-0,060	-0,116	-0,154	-0,077
NQO1	0,315	0,379	0,540	0,269	0,680	1,353	0,224	0,498	0,805
PRDX1	0,069	0,099	0,191	0,017	0,192	0,800	0,070	0,109	0,216
SLC7A11	0,587	0,901	2,352	0,172	1,225	3,855	-0,104	0,024	0,986
SOD3	0,391	-0,332	1,100	-0,322	-0,890	-1,693	1,083	0,093	0,543
SRXN1	0,718	0,916	1,129	0,228	1,229	2,785	0,210	0,523	0,807
TXN	0,208	0,350	0,619	0,122	0,224	0,704	0,122	0,132	0,238
TXNRD1	0,428	0,438	0,912	0,052	0,746	2,414	0,026	0,210	0,690

S5. DCFH-DA Assay – ROS Generation

Figure S2 depicts two positive controls used in the cellular DCFH-DA assay, namely H_2O_2 and tBOOH. While both compounds induced an induction of ROS after 20 min exposure, only 2.5 mM H_2O_2 resulted in a significant increase. Hence, H_2O_2 served as a suitable positive control for the assay.

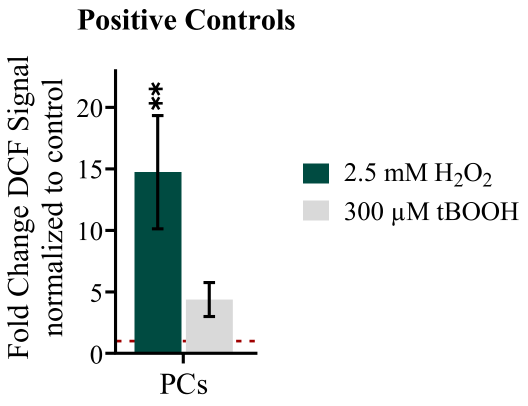

Figure S3. Positive controls (PCs) used for the cellular DCFH-DA assay. BEAS-2B cells were incubated with both compounds for 20 min. Bar chart represents mean of all independent experiments ± sem. An ordinary one-way ANOVA followed by Dunnett's multiple comparison test was applied: $^{**}p \leq 0.01$.

Supplementary Materials: The following supporting information can be downloaded at: https://www.mdpi.com/article/doi/s1, Figure S1: title; Table S1: title; Video S1: title.

Disclaimer/Publisher's Note: The statements, opinions and data contained in all publications are solely those of the individual author(s) and contributor(s) and not of MDPI and/or the editor(s). MDPI and/or the editor(s) disclaim responsibility for any injury to people or property resulting from any ideas, methods, instructions or products referred to in the content.

Academic Editor: Firstname Lastname

Received: date
Revised: date
Accepted: date
Published: date

Copyright: © 2026 by the authors. Submitted for possible open access publication under the terms and conditions of the Creative Commons Attribution (CC BY) license.

https://doi.org/10.3390/xxxxx
